# Immunogenicity of live attenuated Japanese encephalitis SA 14-14-2 vaccine among Sri Lankan children with previous receipt of inactivated JE vaccine

**DOI:** 10.1016/j.vaccine.2016.10.028

**Published:** 2016-11-21

**Authors:** Pushpa Ranjan Wijesinghe, M.R. Nihal Abeysinghe, Sutee Yoksan, Yafu Yao, Benli Zhou, Lei Zhang, Jessica A. Fleming, Anthony A. Marfin, John C. Victor

**Affiliations:** a Epidemiological Unit, Ministry of Healthcare and Nutrition, Colombo, Sri Lanka; b Mahidol University, Bangkok, Thailand; c Chengdu Institute of Biological Products, Chengdu, China; d PATH, Seattle, WA, USA

**Keywords:** Japanese encephalitis vaccines, Clinical trial, Humoral immune response, Secondary immune response, Safety

## Abstract

**Background:**

The performance of live attenuated Japanese Encephalitis SA 14-14-2 vaccine (CD-JEV) among children previously given inactivated mouse brain-derived JE vaccine (IMBV) is unknown. We evaluated the safety and immunogenicity of CD-JEV administered to 2- and 5-year-old children in Sri Lanka.

**Methods:**

In this open-label, single arm trial in the Colombo District of Sri Lanka, generally healthy children 2 and 5 years of age who had previously received two and three doses of IMBV, respectively, were administered one dose of CD-JEV subcutaneously. Participants were monitored for adverse events for one year post-vaccination. Serum neutralizing antibody responses were evaluated pre and 28 and 365 days post-vaccination using JE plaque reduction neutralization test and characterized as the proportion of participants seroconverting. Seroconversion was defined as either reaching a titer considered seroprotective (⩾1:10) among participants with a baseline titer <1:10 or achieving at least a 4-fold rise in titer among participants with a baseline titer ⩾1:10.

**Results:**

Of 305 children given CD-JEV, 294 were included in the primary analysis of immunogenicity. Prior to vaccination, 144/147 (98.0%) 2-year-olds and 146/147 (99.3%) 5-year-olds had seroprotective levels. 28 days post-vaccination, 79/147 [53.7% (95% CI, 45.3–62.0)] 2-year olds and of 60/147 [40.8% (95% CI, 32.8–49.2)] 5-year olds achieved seroconversion. Among 2-year-olds, geometric mean titers (GMTs) rose from 697 to 3175 28 days post-vaccination. Among 5-year-olds, GMTs rose from 926 to 2776. Most adverse reactions were mild, and no serious adverse events were related to study vaccination.

**Conclusion:**

Administration of CD-JEV to these children with pre-existing neutralizing JE antibody titers was safe and resulted in substantial boosting of antibody levels. These results may inform other countries in Asia considering switching from IMBV to now WHO-prequalified CD-JEV vaccine to combat this disease of public health importance.

## 1 Introduction

Japanese encephalitis (JE) is a flavivirus that is transmitted primarily by *Culex* mosquitoes from South Asia to regions of the Western Pacific. JE infection is usually asymptomatic, but symptomatic infections of the brain can be severe, resulting in both permanent brain damage and death. These sequelae, especially among children, and infections occurring among many people during large outbreaks is why JE is a disease of public health concern [1], [2]. Because the virus is zoonotic in regions where it is endemic, vaccines are the primary tool for disease control.

In several countries in Asia, such as Sri Lanka, national immunization programs previously relied on inactivated mouse brain-derived vaccine (IMBV), given in multiple doses (in primary series and as booster doses). New JE vaccines are now available [3]. Switching from IMBV to a new JE vaccine would be programmatically simpler if countries did not need to ensure a completed series with IMBV and could simply give children who had initiated the IMBV series a dose(s) of the new JE vaccine. One new JE vaccine, Chengdu Institute of Biological Products’ (CDIBP) live attenuated SA 14-14-2 JE vaccine (CD-JEV), can be given as a single dose [4]. However, it is unknown how CD-JEV would perform after administration to children with antibodies induced by previous receipt of IMBV. To address this question and provide officials with locally generated immunogenicity and safety data on CD-JEV, the Sri Lanka Ministry of Healthcare and Nutrition initiated this study.

## 2 Methods

### 2.1 Study design

The study was an open label, non-randomized, single-arm trial, conducted in three peri-urban health divisions with low JE endemicity in the District of Colombo. Ethical review was provided by the University of Colombo Faculty of Medicine Ethical Review Committee and PATH’s Research Ethics Committee. Parents or guardians provided written informed consent for all participants. The study, ClinicalTrials.gov NCT00463476, was conducted according to the principles of the Declaration of Helsinki. PATH sponsored the trial and ensured its compliance with Good Clinical Practice (GCP) guidelines.

### 2.2 Participants

Eligible participants were generally healthy children aged 2 years (plus or minus 3 months) and 5 years (plus or minus 3 months) who had previously received all vaccinations recommended under the Sri Lankan childhood immunization schedule according to their age and would attend all planned study visits. Children 2 years of age must have previously received IMBV at the recommended 12 and 13 months of age, and children 5 years of age must have previously received IMBV at the recommended 12, 13, and 24 months of age. Children with a history of acute encephalitis were excluded. Participants were requested to forego other vaccinations from 2 weeks before to 4 weeks after receipt of study vaccine.

### 2.3 Procedures

Participants were consecutively enrolled as consent was obtained. On study day 0, participants received a single 0.5 ml dose of CD-JEV (live attenuated SA 14-14-2 JE vaccine, CDIBP, Chengdu, People’s Republic of China; lot 200611A078-1) delivered subcutaneously in the right upper arm using 23 gauge needles.

Participants were monitored by trained study physicians. During the first 7 days after vaccination, parents completed diary cards for solicited and unsolicited events, grading events according to severity using scales supplied to them by study physicians. Study physicians called or visited the homes of participants 2–3 days after receipt of CD-JEV to review diary cards and assist parents with my severity grading problems. On the 7th day post-vaccination, study physicians visited all homes of study subjects, reviewed diary cards with parents, and performed a physical exam of the participant. At 28 days post-vaccination, parents returned to the study clinic with their child participant, and study physicians interviewed parents about additional unsolicited events and again performed a physical exam. Over the subsequent 10 months, participants were visited or telephoned monthly by study physicians to identify serious adverse events (SAEs). Finally, at one year post-vaccination, parents returned to the study clinic with their child participant for a final visit where physicians performed a final physical exam. During face-to-face visits, ongoing AEs were graded for severity by study physicians. Participants provided blood serum at enrollment and approximately 28 days and one year post-vaccination. Serum samples were frozen at −70 °C and shipped by air on dry ice to the Center for Vaccine Development at Mahidol University in Bangkok for testing.

### 2.4 Outcomes

The primary immunogenicity endpoint was the titer of serum neutralizing antibody to JE. Neutralizing antibodies were measured by plaque reduction neutralization test (PRNT); the method used was a serum dilution, constant virus PRNT_50_ performed in LLC-MK2 cells [5]. Paired serum samples from subjects were tested for antibodies against wild-type Beijing-1 strain, a JE virus belonging to JE virus genotype III, the same genotype as CD-JEV. The end-point for neutralization was the inverse of the dilution at which plaque counts were reduced by 50%, compared with a negative serum control, determined by probit analysis. Seroprotection for JE was defined as a serum neutralizing antibody titer of ⩾1:10 [6].

Safety endpoints included solicited local reactions at the injection site (erythema, induration, and pain), solicited systemic reactions (fever, anorexia, crying, diarrhea, drowsiness, insomnia, irritability, and vomiting), and unsolicited adverse reactions through 7 days post-vaccination. Other unsolicited non-serious adverse events from 8 through 28 days post-vaccination and SAEs at any time during the 1-year study were also collected. SAEs were defined by ICH GCP with the additional criterion that other “important medical events that may not result in death, be life threatening, or require hospitalization may be considered SAEs when, based upon appropriate medical judgment, may jeopardize the subject and may require medical or surgical intervention to prevent one of the outcomes listed by ICH GCP.”

### 2.5 Statistical analyses

The primary objective was to estimate the proportion of 2-year-old and 5-year-old children with seroprotective levels of JE serum neutralizing antibodies 28 days post-vaccination. The secondary objectives were to determine the proportion of children in each age group with seroprotective levels at one year post-vaccination and the geometric mean titer (GMT) of serum neutralizing antibodies to JE at 28 days and one year post-vaccination for both groups. Titers less than the limit of quantification (1:10) were assigned a value of 1:5 for calculation of GMTs. Exact 95% confidence intervals for proportions were calculated under the binomial distribution, and approximate 95% confidence intervals for log GMTs were calculated under the normal distribution. Because WHO recommends that “In people who are seropositive at baseline (i.e. have PRNT_50_ titers ⩾1:10) the primary assessment of immune responses to vaccination would usually be based on proportions achieving substantial increases (e.g. at least a 4-fold rise) in titer after one or more doses of JE vaccine,” [7] antibody rises were also analyzed post-hoc as the proportion of participants with baseline titers <10 who had fold increases in neutralizing antibody between baseline and day 28 post-vaccination of 2.00–2.99, 3.00–3.99, and ⩾4.00. Immunogenicity analyses were conducted on the per-protocol subject population while safety analyses included all vaccinated subjects.

While the study was primarily descriptive, for calculation of required sample size, we assumed the Day 28 post-administration seroprotection would be 90% [8]. Under this assumption, a sample size of 137 evaluable participants in each age group (274 total) would be sufficient to demonstrate that the observed proportion with seroprotective JE antibodies was greater than 80% using one-sided significance levels of 0.025 and a power of at least 90%. This total was inflated to 306 infants to allow for up to 10% loss to follow-up in each group.

## 3 Results

A total of 305 children were enrolled (151 2-years-olds and 154 5-year-olds) between July and October 2007. Baseline characteristics are shown in Table 1. A total of 300 subjects (98.4%) were followed to the end of the study one year later (3 voluntarily withdrew participation, and 2 were lost to follow-up), providing the full 12 months of safety data post-vaccination. Of those enrolled, 294 (96.4%) met criteria for inclusion in the per-protocol analysis (4 were incorrectly included by age, 4 had sera collected outside the specified time range, and 3 were missing serologic results).

At study entry, nearly all children previously vaccinated with IMBV had seroprotective levels of neutralizing antibodies against JE virus (98% of 2-year-olds and 99% of 5-year-olds) (Table 2). GMTs at baseline were high (697 among 2-year-olds and 926 among 5-year-olds). Twenty-eight days post-vaccination with CD-JEV, all children in both age groups had seroprotective antibody levels. Despite high baseline antibody levels, GMTs rose substantially 28 days post-vaccination and persisted near these levels up to 1 year post-vaccination; by age group, GMTs among 2-year-olds 28 days post-vaccination were 3175 (95% CI, 2744–3672) and among 5-year-olds were 2776 (95% CI, 2400–3210). Nearly half of children with titers ⩾10 pre-vaccination with CD-JEV experienced a four-fold or greater rise in titer at 28 days post-vaccination (Table 3). Of 144 2-year olds, 76 (52.8%) had a four-fold or greater rise in their neutralizing antibody titer; of 146 5-year olds, 59 (40.4%) experienced such an increase. Using jointly WHO’s endpoint criteria for primary assessment of immune responses as (a) the proportion of previously seronegative subjects who reach a PRNT_50_ titer against Beijing-1 virus of at least 1:10 after vaccination *or* (b) the proportion of subjects seropositive at baseline who achieve at least a 4-fold rise after vaccination, of 147 2-year olds, 79 [53.7% (95% CI, 45.3–62.0)] met these criteria and of 147 5-year olds, 60 [40.8% (95% CI, 32.8–49.2)] met these criteria.

Of the 305 children previously vaccinated with at least two doses of IMBV and administered CD-JEV, none had immediate reactions to the vaccine (within 30 min). Solicited local reactions were reported almost exclusively during the first three days post-vaccination, and all were graded as less than severe (Table 4). Solicited systemic reactions were generally mild with anorexia, fever, insomnia, and crying being the most commonly reported events during the 7 days post-vaccination (Table 4). Only one subject (0.7%) in each age group had a solicited systemic reaction graded as severe—one 2-year-old with parent-reported irritability and one 5-year-old with parent-reported diarrhea. During the 7 days post-vaccination, parents reported unsolicited adverse reactions in 15 (9.9%) 2-year-olds and 15 (9.7%) 5-year-olds (Table 4). None were graded as severe. At the Day 28 study visit, an additional 100 unsolicited adverse events were reported among 54 (35.8%) 2-year-olds and 89 unsolicited adverse events among 54 (35.1%) 5-year-olds (Table 5). Only one of these events (<1%), diarrhea in a 5-year-old, was graded as severe. Twenty-six serious adverse events were reported among 22 two-year-olds and 9 serious adverse events were reported among 8 five-year-olds up to one year after receipt of CD-JEV. All of these were hospitalizations, and the investigator judged that all SAEs were unrelated to receipt of CD-JEV. There were no deaths, and all participants fully recovered. SAEs occurring through 90 days post-vaccination with CD-JEV are listed in Table 6.

## 4 Discussion

Sri Lanka is similar to other countries that have used IMBVs for the past 20–30 years to control JE but are making a transition to the next generation of JE vaccines. Following large epidemics in 1985–1988, Sri Lanka introduced IMBV to children 1–10 years old. Children in this study had previously received IMBV based on the Beijing-1 JE strain. Although IMBVs require multiple doses to maintain protective antibody titers against JE (a two-dose primary series, first booster dose one year later, and additional booster doses every 4–5 years) [9], no other types of vaccines were available internationally at that time. Although disease incidence was dramatically reduced with introduction and wide use of IMBV over the next two decades, increases in reports of adverse events following immunization (AEFI) negatively impacted vaccine acceptance by parents [10], [11], [12], [13]. In addition, the high cost of IMBV, which comprised nearly three quarters of the Sri Lankan vaccine budget, threatened the financial sustainability of the nationally-funded immunization program [10], [12], [14]. Moreover, inadequate supplies of IMBV in the global market threatened the continuity of Sri Lanka’s established JE vaccination programs.

IMBVs were not widely used in public health immunization programs across JE endemic regions of Asia due to the required multi-dose schedule, the relatively high cost, and limited duration of protection [15], [16]. In 2006 WHO recommended that IMBVs be gradually replaced by alternative JE vaccines [16]. For Sri Lanka, this switch was prompted when IMBV was not available in both 2007 and 2008 [14], leaving a large cohort of children unvaccinated or partially vaccinated. The Sri Lanka Ministry of Healthcare and Nutrition identified CD-JEV as a low-cost alternative to IMBV [12], [17]. However, before recommending its use for subsequent JE vaccine doses in children who had previously received IMBV, the Sri Lankan National Advisory Committee on Communicable Diseases requested clinical trial data to provide evidence of vaccine safety and immunogenicity in the Sri Lankan population.

This study answers two important questions for JE vaccination programs considering transition from older JE vaccines to the next generation of JE vaccines or boosting primary vaccination series with CD-JEV. First, high titers of neutralizing antibody to JE resulting from vaccination with IMBV do not appear to interfere with the boosting effect of CD-JEV. Regardless of the age of the vaccine recipient or the number of IMBV doses previously received, the GMT greatly increased and remained elevated one year later. Second, although this was a small trial, the safety profile of CD-JEV was good, with solicited or unsolicited adverse reactions and adverse events during the first month after vaccination rarely being graded as “severe.”

Specifying the degree of improved immune protection resulting from the CD-JEV booster is difficult because of the high seroprotection rate noted at baseline and because specific changes in cell-mediated immunity resulting from use of a live vaccine were not measured in this study. In this study, the overall seroprotection rate increased from 99% at baseline to 100% 28 days after vaccination. In a similar study in a low JE prevalence area in Korea, only 16/25 (64%) of children who previously received a two-dose primary series with IMBV had a measurable JE neutralizing antibody titer ⩾1:10 approximately 7 months later [18]. In this same study, among another 16 children with history of previous receipt of IMBV or baseline detectable JE neutralizing antibodies, 100% were seroprotected after a single dose of CD-JEV, and among those with documented history of previous receipt of IMBV the GMT post-vaccination with CD-JEV was 3378. Similar results have been reported from other countries [19], [20]. The reasons for the high seroprotection rates at baseline in our study are unknown.

In this study, there was a large increase in neutralizing antibody GMTs following booster vaccination and, overall, about 45% of children had at least a four-fold rise in neutralizing antibody titer. One year later, the GMTs had not declined. Given the high seroprotection rate at baseline in our study, the increase in GMTs at 28 days and one year after the booster dose of CD-JEV appear to be a more meaningful measure of CD-JEV’s boosting effect. These increases support the hypothesis that, despite the presence of preexisting neutralizing antibodies, CD-JEV will induce a secondary immune response after previous receipt of IMBV.

A number of clinical trials and observational studies have shown CD-JEV to be safe and the Global Advisory Committee on Vaccine Safety of the World Health Organization has acknowledged its excellent safety profile when given in a primary series [2], [8], [21], [22], [23]. Our study adds to the safety profile in children previously vaccinated with IMBV. Despite the very high baseline anti-JE antibody titers in these children, active AEFI surveillance showed that AEFI consistent with antibody-mediated Type I, II, or III hypersensitivity reactions were very rare in the year following the booster dose. No serious adverse events were attributed to CD-JEV administration.

Our study used a “before and after design”, and as such, did not include a control group for comparison. This design has the inherent limitation of reduced internal validity and given the possibility of potentiation of the immune response due to natural boosting by circulating JE and dengue viruses in a study area endemic to both, inclusion of a control group would have helped quantify changes in immunogenicity due to CD-JEV. This was not possible, as withholding JE vaccine from a control group in a JE endemic area would be unethical, and the unavailability of the IMBV in Sri Lanka precluded the use of a comparator group using that vaccine. Similarly, while we believe JE endemicity was low in our study population, we also did not evaluate pre- or post-vaccination sera for the presence of antibodies to other flaviviruses, such as dengue viruses. We utilized the WHO-recommended PRNT_50_ to measure JE neutralizing antibodies, as did the researchers in the aforementioned Korean study, but a JE PRNT_90_ might have helped to differentiate baseline JE antibodies from cross-reaction with antibodies due to exposure to other flaviviruses such as dengue [24]. Despite these limitations, the data from this study adds to the evidence that CD-JEV is safe and immunogenic in children who have been primed with at least two doses of inactivated JE vaccine. In July 2009, the Sri Lanka’s National Advisory Committee on Communicable Diseases approved the use of CD-JEV in children who had previously received inactivated JE vaccine and recommended its incorporation into the routine EPI schedule [12], [25].

While several new JE vaccines are now available, CD-JEV has the advantages of being given as a single dose, a reduced price for the public sector, and demonstrated protection. Since 2013, Gavi, The Vaccine Alliance has committed to provide financial support to eligible countries interested in conducting JE vaccination campaigns prior to introduction of sustainable routine infant and child JE vaccination. Currently, nearly a dozen Asian countries, including Sri Lanka, have national or subnational JE vaccination programs. Five currently, Camboda, China, India, Nepal and Sri Lanka use CD-JEV vaccine [2]. With Gavi support now available and the safety, effectiveness, and feasibility of CD-JEV now established, other countries in Asia and the Western Pacific have the opportunity to introduce CD-JEV to combat this disease of public health importance.

## Contributors and role of the funding source

MRNA, PRW, and JCV contributed to the study design. MRNA and PRW supervised the implementation of the study at the sites. YS supervised the conduct of all laboratory assays. JCV and PRW verified protocol-stated statistical analyses that were conducted by a statistical consultant; JCV conducted post-hoc analyses. All authors had full access to the data and results. MRNA, PRW, JAF, AAM, and JCV participated in drafting of this manuscript or in critically revising the draft. All authors reviewed and approved the final version of the manuscript. The corresponding author had final responsibility for the decision to submit for publication. Investigators and their institution were funded by PATH’s Japanese Encephalitis Project, under a grant from the Bill & Melinda Gates Foundation, #28658. CDIBP donated CD-JEV vaccine for the study, and their staff approved of the study but held only observer/advisor status. PATH acted as the regulatory sponsor, and PATH and a PATH-designated CRO were responsible for study initiation, clinical monitoring, pharmacovigilance, data management, data analysis, and reporting.

## Conflict of interest

Y. Yao, B. Zhou, and L. Zhang are employees of CDIBP. J. Fleming, A. Marfin, and J. Victor are employees of PATH, which has received a grant from the Bill & Melinda Gates Foundation to ensure quality, supply, and optimal programmatic use of CD-JEV in low-resource populations in Asia. No other conflicts of interest were identified.

## Figures and Tables

**Table 1:** Baseline demographic characteristics of enrolled participants.

		2-year-olds (n = 151)	5-year-olds (n = 154)
Sex, n (%)	Female	79 (52.3%)	76 (49.4%)
	Male	72 (47.7%)	78 (50.6%)

Ethnicity, n (%)	Sinhalese	142 (94.0%)	151 (98.1%)
	Sri Lankan Tamil	4 (2.6%)	0 (0.0%)
	Sri Lankan Moor	5 (3.3%)	2 (1.3%)
	Malay	0 (0.0%)	1 (0.6%)

Age (months), mean (SD)		24.5 (1.4)	60.4 (1.6)

Weight (kg), mean (SD)		10.9 (1.5)	16.2 (2.7)

Height (cm), mean (SD)		83.7 (4.6)	106.3 (5.5)

**Table 2:** Proportions seroprotected^a^ and geometric mean titers of JE serum neutralizing antibodies among children receiving CD-JEV after previous receipt of two (2-year-olds) or three doses (5-year-olds) of inactivated mouse-brain-derived JE vaccine.

Time post-vaccination	2-year-olds			5-year-olds		
	Freq	Percent (95% CI)	GMT (95% CI)	Freq	Percent (95% CI)	GMT (95% CI)
Day 0	144/147	98.0 (94.2, 99.6)	697 (551, 883)	146/147	99.3 (96.3, 100.0)	926 (733, 1170)
Day 28	147/147	100.0 (97.5, 100.0)	3175 (2744, 3672)	147/147	100.0 (97.5, 100.0)	2776 (2400, 3210)
1 year	145/145	100.0 (97.5, 100.0)	3170 (2674, 3758)	143/144	99.3 (96.2, 100.0)	2585 (2131, 3136)

a Seroprotection was defined as neutralizing antibody titer ⩾1:10.

**Table 3:** Proportions of children with baseline neutralizing antibody titers ⩾10 experiencing fold-level increases in JE neutralizing antibodies from pre-vaccination to 28 days post-vaccination with CD-JEV after previous receipt of two (2-year-olds) or three doses (5-year-olds) of inactivated mouse-brain-derived JE vaccine.

Increase factor	2-year-olds (n = 144)		5-year-olds (n = 146)	
	Freq	Percent (95% CI)	Freq	Percent (95% CI)
<2.00	42	29.2% (21.9, 37.3)	63	43.2% (35.0, 51.6)
2.00–2.99	12	8.3% (4.4%, 14.1)	15	10.3% (5.9%, 16.4%)
3.00–3.99	14	9.7% (5.4, 15.8)	9	6.2% (2.9, 11.4)
⩾4.00	76	52.8% (44.3, 61.2)	59	40.4% (32.4, 48.8)

**Table 4:** Summary of solicited and unsolicited adverse reactions during the period 7 days post-vaccination.

	Severity	2-year-olds (n = 151)		5-year-olds (n = 154)	
		n^a^	% (95% CI)	n^a^	% (95% CI)
*Solicited local reactions (days 0–3)*					
Any	Any	31	20.5 (14.4; 27.9)	46	29.9 (22.8, 37.8)
	Severe	0	0.0 (0.0, 2.4)	0	0.0 (0.0, 2.4)

Erythema	Any	7	4.6 (1.9, 9.3)	7	4.6 (1.9; 9.1)
	Severe	0	0.0 (0.0, 2.4)	0	0.0 (0.0, 2.4)

Induration	Any	8	5.3 (2.3, 10.2)	9	5.8 (2.7; 10.8)
	Severe	0	0.0 (0.0, 2.4)	0	0.0 (0.0, 2.4)

Pain	Any	28	18.5 (12.7, 25.7)	44	28.6 (21.6; 36.4)
	Severe	0	0.0 (0.0, 2.4)	0	0.0 (0.0, 2.4)

*Solicited Systemic reactions (days 0–7)*					
Any	Any	44	29.1 (22.0, 37.1)	38	24.7 (18.1, 32.3)
	Severe	1	0.7 (0.0, 3.6)	1	0.7 (0.0, 3.6)

Fever	Any	12	8.0 (4.2, 13.5)	16	10.4 (6.1, 16.3)
	Severe	0	0.0 (0.0, 2.4)	0	0.0 (0.0, 2.4)

Anorexia	Any	28	18.5 (12.7, 25.7)	16	10.4 (6.1, 16.3)
	Severe	0	0.0 (0.0, 2.4)	1	0.7 (0.0, 3.6)

Crying	Any	15	9.9 (5.7, 15.9)	8	5.2 (2.3, 10.0)
	Severe	0	0.0 (0.0, 2.4)	0	0.0 (0.0, 2.4)

Diarrhea	Any	5	3.3 (1.1, 7.6)	1	0.7 (0.0, 3.6)
	Severe	0	0.0 (0.0, 2.4)	1	0.7 (0.0, 3.6)

Tiredness	Any	9	6.0 (2.8, 11.0)	5	3.3 (1.1, 7.4)
	Severe	0	0.0 (0.0, 2.4)	0	0.0 (0.0, 2.4)

Insomnia	Any	13	8.6 (4.7, 14.3)	5	3.3 (1.1, 7.4)
	Severe	0	0.0 (0.0, 2.4)	0	0.0 (0.0, 2.4)

Irritability	Any	6	4.0 (1.5, 8.5)	5	3.3 (1.1, 7.4)
	Severe	1	0.7 (0.0, 3.6)	0	0.0 (0.0, 2.4)

Vomiting	Any	6	4.0 (1.5, 8.5)	6	3.9 (1.4, 8.3)
	Severe	0	0.0 (0.0, 2.4)	0	0.0 (0.0, 2.4)

*Unsolicited reactions (days 0–7)*					
Any	Any	15	9.9 (5.7, 15.9)	15	9.7 (5.6, 15.6)
	Severe	0	0.0 (0.0, 2.4)	0	0.0 (0.0, 2.4)

a Number of subjects experiencing event during specified time-period.

**Table 5:** Unsolicited adverse events (total and among select organ classes) among subjects Days 8–28 post-vaccination with CD-JEV.

MedDRA SYSTEM ORGAN CLASS Preferred Term	2-year-olds (n = 151)				5-year-olds (n = 154)			
	Subjects^a^		Events		Subjects^a^		Events	
	n	%	n	%	n	%	n	%
ANY NON-SERIOUS ADVERSE EVENT	54	35.8%	100	100%	54	35.1%	89	100%
EYE DISORDERS	2	1.3%	2	2.0%	1	0.6%	1	1.1%
Conjunctivitis	1	0.7%	1	1.0%	0	0.0%	0	0.0%
Eye swelling	0	0.0%	0	0.0%	1	0.6%	1	1.1%
Ocular hyperaemia	1	0.7%	1	1.0%	0	0.0%	0	0.0%
GENERAL DISORDERS AND ADMINISTRATION SITE CONDITIONS	16	10.6%	16	16.0%	17	11.0%	17	19.1%
Pyrexia	16	10.6%	16	16.0%	17	11.0%	17	19.1%
NERVOUS SYSTEM DISORDERS	2	1.3%	2	2.0%	0	0.0%	0	0.0%
Febrile convulsion	2	1.3%	2	2.0%	0	0.0%	0	0.0%
SKIN AND SUBCUTANEOUS TISSUE DISORDERS	4	2.6%	4	4.0%	4	2.6%	6	6.7%
Blister	1	0.7%	1	1.0%	1	0.6%	1	1.1%
Pruritus	0	0.0%	0	0.0%	1	0.6%	1	1.1%
Rash	0	0.0%	0	0.0%	1	0.6%	1	1.1%
Rash papular	0	0.0%	0	0.0%	1	0.6%	1	1.1%
Rash pruritic	1	0.7%	1	1.0%	1	0.6%	1	1.1%
Urticaria	2	1.3%	2	2.0%	1	0.6%	1	1.1%

a Each subject is counted once per “system organ class/preferred term”.

**Table 6:** Serious adverse events among all subjects during the 90 days post-vaccination.

Subject number	Event	Onset day post-vaccination	Severity^a^
*2-year-olds*			
103–042	Lower respiratory tract infection	5	Moderate
201–062	Urticaria	19	Moderate
203–058	Viral infection	20	Moderate
103–042	Lower respiratory tract infection	24	Moderate
201–007	Febrile convulsion	27	Moderate
202–039	Lower respiratory tract infection	36	Moderate
202–039	Tonsillitis	36	Moderate
203–010	Asthma	46	Moderate
203–020	Penile swelling	47	Moderate
203–041	Viral infection	48	Mild
201–054	Viral infection	68	Moderate
201–040	Urticaria	84	Moderate

*5-year-olds*			
202–007	Thermal burn	2	Mild
202–037	Enterobiasis	21	Moderate
203–028	Upper respiratory tract infection	81	Mild
302–039	Viral infection	89	Moderate

^a^ Severity was graded as follows:

Mild: events require minimal or no treatment and do not interfere with the child’s functioning.

Moderate: events result in a low level of concern with therapeutic measures. Moderate events may cause some interference with functioning.

Severe: events interrupt the child’s functioning and may require systemic drug therapy or other treatment. Severe events are usually incapacitating.

Life threatening: any adverse drug experience that places the child, in the view of the investigator, at immediate risk of death from the reaction *as it occurred*. (The investigator should *not* include a reaction that had it occurred in a more severe form, might have caused death.)
